# Serelaxin treatment reverses vascular dysfunction and left ventricular hypertrophy in a mouse model of Type 1 diabetes

**DOI:** 10.1038/srep39604

**Published:** 2017-01-09

**Authors:** Hooi Hooi Ng, Chen Huei Leo, Darnel Prakoso, Chengxue Qin, Rebecca H. Ritchie, Laura J. Parry

**Affiliations:** 1 School of BioSciences, The University of Melbourne, Parkville, Victoria 3010, Australia; 2 Heart Failure Pharmacology, Baker IDI Heart & Diabetes Institute, Melbourne, Victoria 3004, Australia

## Abstract

Serelaxin prevents endothelial dysfunction in the mouse aorta *ex vivo* and inhibits apoptosis in cardiomyocytes under acute hyperglycaemia. Less is known about the effects of serelaxin in an *in vivo* mouse model of diabetes. Therefore, we tested the hypothesis in streptozotocin (STZ)-treated mice that serelaxin is able to reverse diabetes-induced vascular dysfunction and cardiac remodelling. Mice were divided into citrate buffer + placebo, STZ + placebo and STZ + serelaxin (0.5 mg/kg/d, 2 weeks) groups. After 12 weeks of diabetes, sensitivity to the endothelium-dependent agonist acetylcholine (ACh) was reduced in the mesenteric artery. This was accompanied by an enhanced vasoconstrictor prostanoid contribution and a decrease in endothelium-derived hyperpolarisation (EDH)-mediated relaxation. Serelaxin restored endothelial function by increasing nitric oxide (NO)-mediated relaxation but not EDH. It also normalised the contribution of vasoconstrictor prostanoids to endothelial dysfunction and suppressed diabetes-induced hyper-responsiveness of the mesenteric artery to angiotensin II. Similarly, diabetes reduced ACh-evoked NO-mediated relaxation in the aorta which was reversed by serelaxin. In the left ventricle, diabetes promoted apoptosis, hypertrophy and fibrosis; serelaxin treatment reversed this ventricular apoptosis and hypertrophy, but had no effect on fibrosis. In summary, serelaxin reversed diabetes-induced endothelial dysfunction by enhancing NO-mediated relaxation in the mouse vasculature and attenuating left ventricular hypertrophy and apoptosis.

Diabetes is associated with cardiovascular complications such as endothelial dysfunction and cardiomyopathy. Endothelial dysfunction is characterised by impaired endothelium-dependent relaxation in blood vessels of both human^1^ and experimental animal models^2^,^3^ of diabetes. Endothelium-dependent relaxation is mediated by three major signals, namely nitric oxide (NO), prostacyclin (PGI_2_) and endothelium-derived hyperpolarisation (EDH). Several lines of evidence suggest that diabetes reduces EDH-mediated relaxation in mesenteric arteries^4^,^5^, carotid arteries^6^ and retinal arterioles^7^ of streptozotocin (STZ)-induced animals. However, few studies have reported an augmentation in EDH-mediated relaxation in diabetes in the aorta^8^,^9^. Under physiological conditions, NO dampens the role of EDH, but when there is an increase of superoxide production caused by hyperglycaemia, the role of EDH in mediating vasorelaxation becomes more apparent as opposed to NO in large vessels. Apart from EDH and NO, PGI_2_ is also involved in the preservation of vasorelaxation in diabetes^8^,^10^. For instance, endothelial dysfunction is prevented by an upregulation of cyclooxygenase (COX)−2 expression and activity in the mesenteric arteries of STZ-induced diabetic mice^11^.

Diabetes-induced endothelial dysfunction has been linked with the pathogenesis of cardiomyopathy and heart failure. Diabetic cardiomyopathy is characterised by impaired myocardial relaxation, left ventricular (LV) fibrosis and hypertrophy, increased apoptosis and oxidative stress^12^. LV hypertrophy often precedes the morphological manifestation of diabetic cardiomyopathy, as evidenced by excess LV mass, which subsequently leads to a stiffer ventricle^13^. Indeed, we have previously demonstrated an increase in cardiomyocyte size that is associated with an upregulation of anti-hypertrophic genes such as natriuretic peptide type B (BNP), β-myosin heavy chain and atria natriuretic peptide (ANP) in the LV of diabetic animals^14^,^15^,^16^,^17^.

Currently, there are many combination therapies to treat diabetes. However, the primary goals of these therapies are to achieve a good glycaemic control, which is insufficient to reduce diabetes-related cardiovascular mortality^18^. Thus, it is critical to look for novel therapeutic agents that can reverse cardiovascular complications associated with diabetes.

Relaxin (RLX) is a 6 kDa peptide hormone that has pleiotropic effects in the vascular system^19^. It mediates its actions through its major receptor, relaxin/insulin-like family peptide receptor 1 (RXFP1), which is localised in the endothelial and vascular smooth muscle cells in both arteries and veins^20^,^21^. Relaxin infusion for 48 hours increases bradykinin (BK)-evoked NO-mediated relaxation, basal NO synthase (NOS) activity and endothelial NOS (eNOS) protein expression, as well as increases both NO and COX-2 derived PGI_2_-mediated relaxation at 72 hours post infusion in healthy rat mesenteric arteries^22^. We recently showed that relaxin co-treatment for 72 hours stimulates PGI_2_ production in the mouse aorta under high glucose conditions *ex vivo*^23^. The positive effects of relaxin treatment have also been reported in the heart as evidenced by attenuation of myocardial injury and preservation of cardiac function by alleviating cardiac fibrosis and ventricular dysfunction in an isoproterenol-induced myocardial ischemic rat model^24^. In the heart of spontaneously hypertensive rats (SHRs), relaxin treatment for two weeks ameliorates ventricular hypertrophy and fibrosis by reducing collagen content, leading to improved cardiac function^25^. Furthermore, relaxin also reverses atrial fibrillation by reducing fibrosis and hypertrophy, which is associated with an increase in Na^+^ current density^26^,^27^. The cardioprotective actions of relaxin are, at least in part, due to its ability to upregulate Notch signalling and inhibit the transforming growth factor-β (TGF-β)/Smad3 pathway, thereby preventing cardiac fibroblast-myofibroblast transition and limiting fibrosis^28^. The anti-hypertrophic and anti-apoptotic actions of relaxin are exerted via its action in reducing oxidative stress involving the extracellular signal–regulated kinase (ERK) and protein kinase B (Akt) signalling pathways in cultured neonatal rat cardiomyocytes^29^. In a mouse model of Type 2 diabetes, relaxin treatment reverses muscle insulin resistance and increases endothelium-dependent relaxation in the aorta of mice fed a high fat diet^30^. There is, however, a lack of evidence that relaxin treatment is effective in reversing a broader range of diabetes-induced cardiovascular complications, particularly in a model of Type 1 diabetes. Therefore, in the present study, we tested the hypothesis that *in vivo* recombinant human relaxin-2 (serelaxin) treatment reverses vascular dysfunction in the mesenteric artery and aorta, as well as ameliorates LV remodelling in the STZ-induced diabetes mouse model.

## Results

### Systemic characteristics following 12-weeks of STZ-induced diabetes

Blood glucose levels in STZ-induced diabetic mice were significantly (*P *< 0.0001) higher than control mice over the 12-week period, and serelaxin treatment in diabetic mice had no effect on blood glucose levels (Supplementary Fig. 1). Glycated haemoglobin (HbA_1c_) and plasma osmolality were significantly (*P* < 0.0001) lower in control mice in comparison to diabetic mice (Table 1). Body, whole heart, left and right ventricle weights of control mice were significantly higher compared to diabetic mice (Table 1). Serelaxin treatment for two weeks (which yielded plasma serelaxin levels of 93 ± 16 ng/mL) had no effect on HbA_1c_, plasma osmolality, body, whole heart, left and right ventricle weight of diabetic mice (Table 1). Atria weight and tibia length were not altered between treatment groups (Table 1).

### Effects of serelaxin on vascular smooth muscle reactivity to vasoconstrictors in the mesenteric arteries

**Figure 1:**
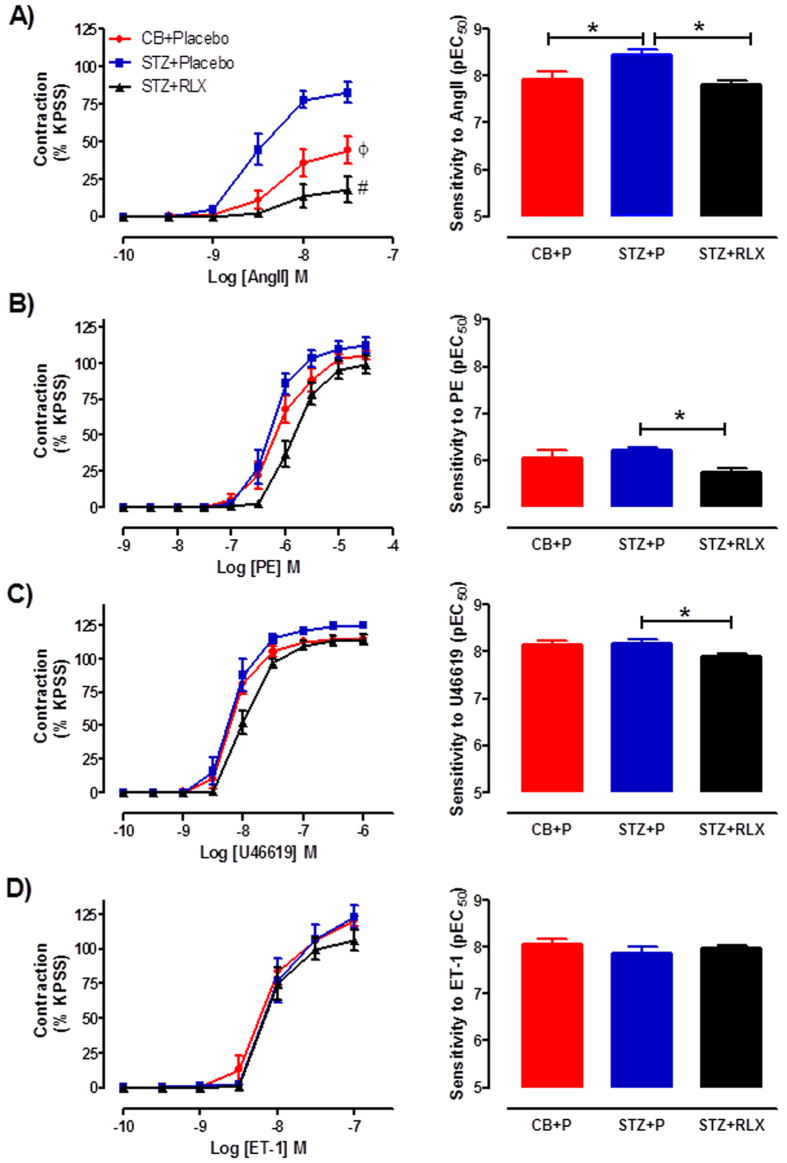
Cumulative dose-response curves and sensitivity to (**A**) angiotensin II, (**B**) phenylephrine, (**C**) U46619 and (**D**) endothelin-1 in the mesenteric arteries of CB + Placebo (●), STZ + Placebo (■) and STZ + RLX (▲) treated mice. *n* = 6–13 per group. Data (mean ± SEM) are expressed as % KPSS contraction. *pEC_50_ significantly (*P* < 0.05) different to STZ + Placebo. ^ϕ^C_max_ significantly (*P* < 0.01) different to STZ + Placebo. ^#^C_max_ significantly (*P* = 0.0001) different to STZ + Placebo.

Diabetes significantly increased the sensitivity (*P* = 0.031) and maximal contraction (*P* = 0.009) to angiotensin II (Ang II) in the mesenteric arteries of STZ + Placebo compared to CB + Placebo treated mice (Fig. 1A). Serelaxin significantly reduced the sensitivity (*P* = 0.014) and maximal contraction (*P* = 0.0001) to Ang II in diabetic mice (Fig. 1A). Diabetes had no effect on the sensitivity and maximal contraction to phenylephrine (PE) and U46619. However, serelaxin significantly reduced the sensitivity but not maximal contraction to both PE (*P* = 0.015) and U46619 (*P* = 0.017) in the mouse mesenteric arteries compared to STZ + Placebo treated mice (Fig. 1B,C). There was no effect of diabetes and serelaxin treatment on endothelin-1 (ET-1)-induced contraction in the mesenteric arteries, indicated by similar sensitivity and maximal contraction to ET-1 (Fig. 1D).

### Effects of serelaxin on endothelial and smooth muscle function in the mesenteric arteries

**Figure 2:**
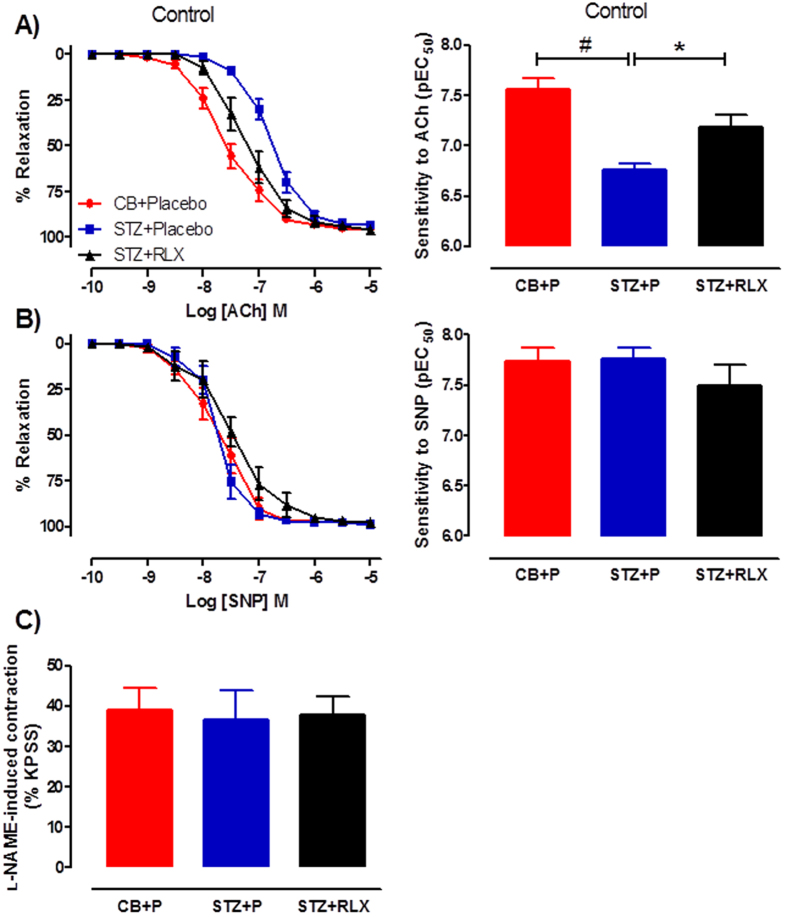
Cumulative dose-response curves and sensitivity to (**A**) ACh and (**B**) SNP in the mesenteric arteries of CB + Placebo (●), STZ + Placebo (■) and STZ + RLX (▲) treated mice. (**C**) Basal NOS activity in the mesenteric arteries of CB + Placebo, STZ + Placebo and STZ + RLX treated mice pre-contracted with U46619 to similar level before the addition of NOS inhibitor, L-NAME (200 μM). *n* = 6–13 per group. Data (mean ± SEM) are expressed as % U46619 or %KPSS contraction. pEC_50_ significantly (^#^*P* < 0.0001, *P < 0.05) different to STZ + Placebo.

Diabetes significantly (*P *< 0.0001) reduced the sensitivity but not maximal relaxation to the endothelium-dependent vasodilator, acetylcholine (ACh) in the mesenteric arteries (Fig. 2A), indicating endothelial dysfunction. Serelaxin significantly (*P* = 0.013) restored the sensitivity to ACh (Fig. 2A), reversing diabetes-induced endothelial dysfunction in the mouse mesenteric arteries. There was no evidence of diabetes-induced vascular smooth muscle dysfunction as indicated by similar relaxation responses to the endothelium-independent vasodilator, sodium nitroprusside (SNP), between the three groups (Fig. 2B). Serelaxin’s ability to reverse diabetes-induced endothelial dysfunction was not due to an alteration in basal NOS activity assessed by the addition of L-NAME to sub-maximally (~25% KPSS) contracted arteries, since there was no significant difference between the three groups (Fig. 2C).

### Effects of serelaxin on the mechanisms of endothelium-dependent relaxation in the mesenteric arteries

**Figure 3:**
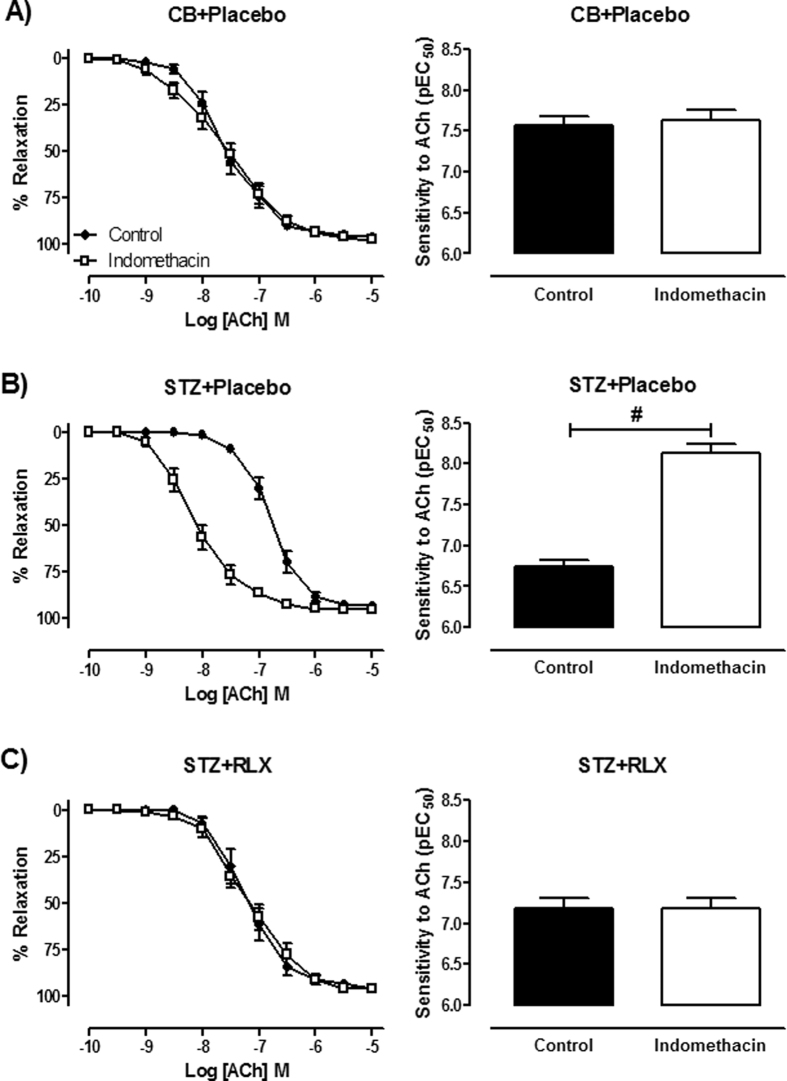
Cumulative dose-response curves and sensitivity to ACh in the absence (control, ●) and presence of the COX inhibitor, indomethacin (1 μM, □) in the mesenteric arteries of (**A**) CB + Placebo, (**B**) STZ + Placebo and (**C**) STZ + RLX treated mice. *n* = 9–13 per group. Data (mean ± SEM) are expressed as % U46619 contraction. ^#^pEC_50_ significantly (*P* < 0.0001) different to control.

To investigate the role of prostanoids in diabetes-induced endothelial dysfunction, responses to ACh were tested in the presence of the COX inhibitor, indomethacin. In the presence of indomethacin, sensitivity and maximal relaxation to ACh were similar to control in CB + Placebo mice (Fig. 3A). However, there was a significant (*P *< 0.0001) increase in sensitivity but not maximal relaxation to ACh in the presence of indomethacin compared to control (Fig. 3B), suggesting the possible increase of vasoconstrictor prostanoids in the mesenteric arteries of STZ + Placebo mice. This effect was normalised by serelaxin, as indicated by similar sensitivity and maximal relaxation to ACh in the presence of indomethacin when compared to control (Fig. 3C).

**Figure 4:**
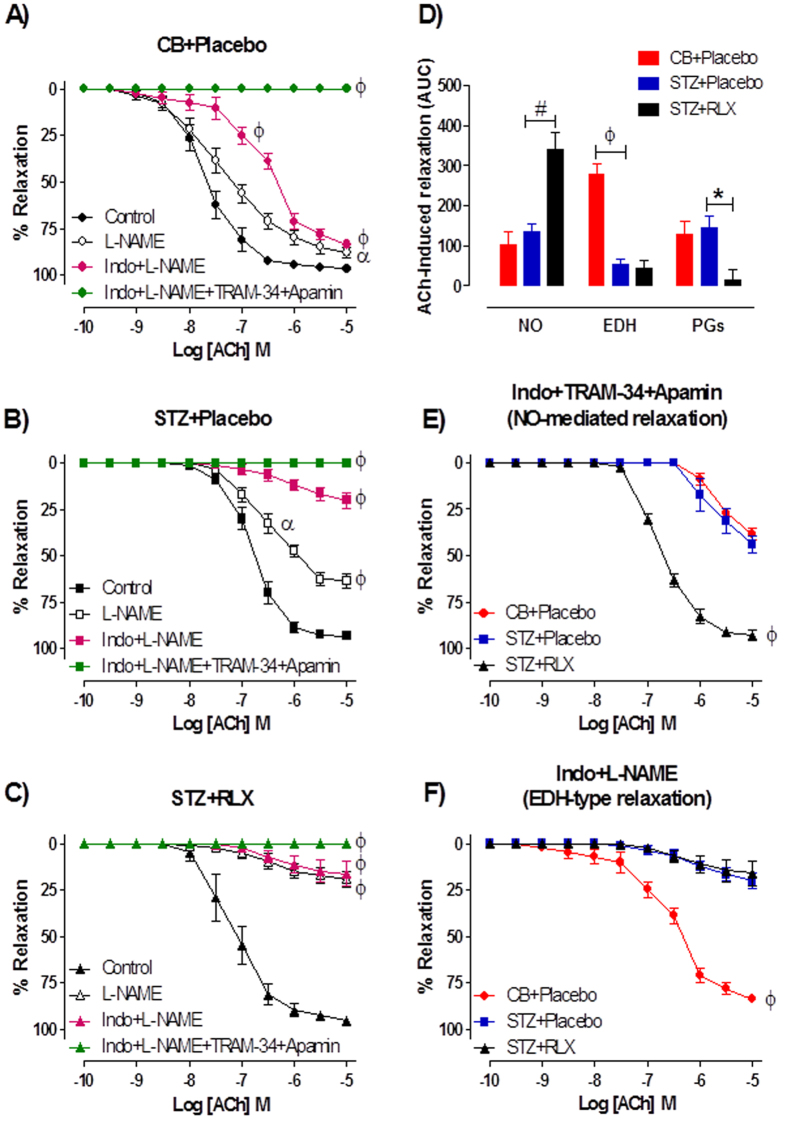
Cumulative dose-response curves to ACh in the absence (control) or presence of the NOS inhibitor, L-NAME (200 μM), indomethacin (1 μM) + L-NAME or the combination of TRAM-34 (1 μM) + apamin (1 μM) + indomethacin + L-NAME in the mesenteric arteries of (**A**) CB + Placebo, (**B**) STZ + Placebo and (**C**) STZ + RLX treated mice. (**D**) Area under curve of ACh-induced relaxation reveals the relative contribution of NO, EDH and PGI_2_ in the mesenteric arteries of CB + Placebo, STZ + Placebo and STZ + RLX treated mice. Cumulative dose-response curves to ACh in the presence of (**E**) indomethacin + TRAM-34 + apamin and (**F**) indomethacin + L-NAME in the mesenteric arteries of CB+Placebo (●), STZ+Placebo (■) and STZ+RLX (▲) treated mice. *n* = 9–12 per group. Data (mean ± SEM) are expressed as % U46619 contraction. Significantly (^ϕ^*P* < 0.0001, ^α^*P* < 0.05, ^#^*P* < 0.001, ^*^P = 0.009) different to STZ+Placebo (comparison across groups) or control (comparison within group).

To investigate the relative contributions of NO, EDH and PGI_2_-mediated relaxation in the mesenteric arteries, responses to ACh were tested in the presence of the NOS inhibitor, L-NAME or the combination of L-NAME and indomethacin or together with the intermediate and small conductance Ca^2+^ activated K^+^ channel blockers, TRAM-34 and apamin. In CB + Placebo treated mice, the sensitivity to ACh was significantly (*P *< 0.0001) reduced in the presence of indomethacin + L-NAME compared to control, but was not affected by the presence of L-NAME alone (control pEC_50_ = 7.67 ± 0.12; L-NAME pEC_50_ = 7.40 ± 0.15, *P* = 0.248) (Fig. 4A). In contrast, the maximal relaxation to ACh was significantly attenuated by the presence of both L-NAME (*P* = 0.020) and indomethacin + L-NAME (*P* < 0.001) (Fig. 4A) in the mesenteric arteries of CB + Placebo mice. In STZ + Placebo and STZ + RLX treated mice, the sensitivity (*P* = 0.043) and maximal relaxation (*P* < 0.0001) to ACh were significantly reduced in the presence of L-NAME and indomethacin + L-NAME in comparison to control (Fig. 4B,C). In all groups, the presence of indomethacin + L-NAME + TRAM-34 + apamin completely abolished ACh-evoked relaxation (Fig. 4A,B,C). Area under curve (AUC) analysis of ACh-response curves revealed that diabetes had no effect on the relative contribution of NO in ACh-evoked relaxation, but it was significantly (*P* < 0.001) increased in the mesenteric arteries of STZ + RLX mice compared to STZ + Placebo mice (Fig. 4D). Diabetes significantly (*P* < 0.0001) decreased the contribution of EDH-type relaxation in the mesenteric arteries of STZ + Placebo mice and serelaxin failed to restore this deficit in diabetic mice (Fig. 4D). Interestingly, there was a significant (*P* = 0.009) reduction in the contribution of vasodilator prostanoids, likely to be PGI_2_ in ACh-evoked relaxation in STZ + RLX mice, assessed by the difference in AUC between L-NAME and indomethacin + L-NAME (Fig. 4D).

The responses to ACh were also tested in the presence of indomethacin + TRAM-34 + apamin to investigate NO-mediated relaxation. Responses to ACh were almost completely abolished in the mesenteric arteries of CB + Placebo and STZ + Placebo mice (Fig. 4E). Serelaxin significantly (*P* < 0.0001) increased the maximal relaxation to ACh in diabetic mice (Fig. 4E), indicating an enhanced NO-mediated relaxation. Similarly, EDH-type relaxation was determined in the presence of indomethacin + L-NAME. The relative contribution of EDH was significantly (*P* < 0.0001) reduced in the mesenteric arteries of STZ + Placebo mice as compared to CB + Placebo mice, indicated by attenuated responses to ACh in the presence of indomethacin + L-NAME (Fig. 4F). Serelaxin failed to restore EDH-mediated relaxation in the mesenteric arteries of diabetic mice (Fig. 4F).

### Effects of serelaxin on endothelial function in the abdominal aortae

**Figure 5:**
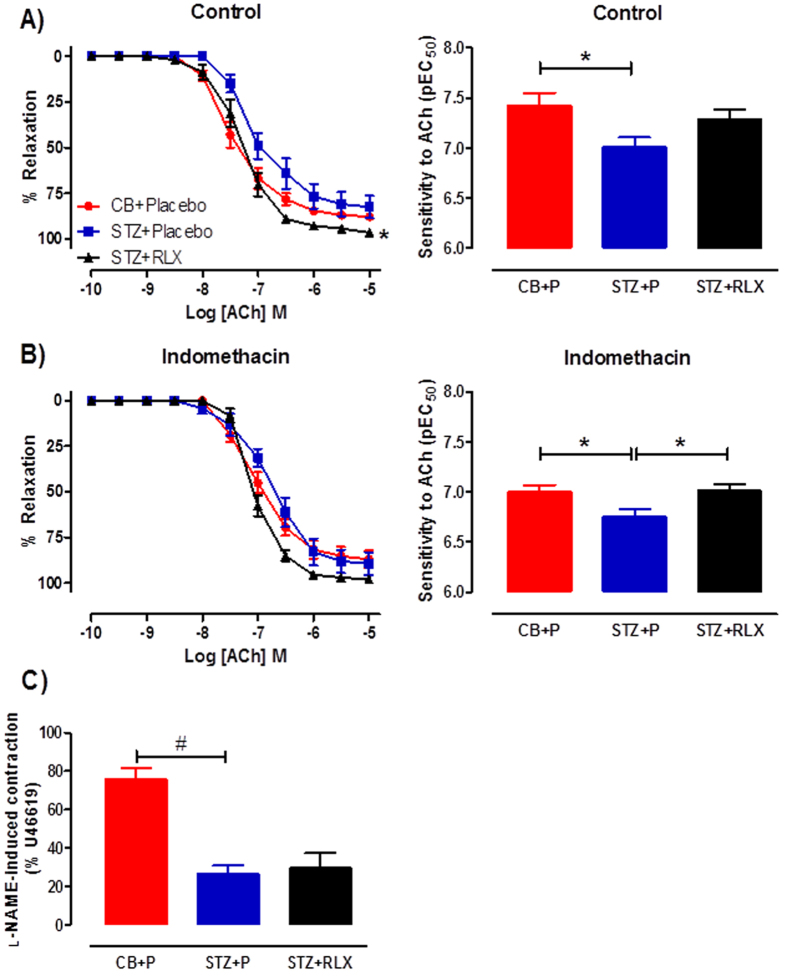
Cumulative dose-response curves and sensitivity to ACh in the (**A**) absence (control) or (**B**) presence of indomethacin in the aortae of CB+Placebo (●), STZ+Placebo (■) and STZ+RLX (▲) treated mice. (**C**) Basal NOS activity in the aortae of CB+Placebo, STZ+Placebo and STZ+RLX treated mice pre-contracted with U46619 to similar level before the addition of NOS inhibitor, L-NAME (200 μM). *n* = 5–6 per group. ^*^pEC_50_/ R_max_ significantly (*P* < 0.05) different to STZ+Placebo. ^#^significantly (*P* = 0.0001) different to STZ+Placebo.

Diabetes significantly (*P* = 0.029) reduced the sensitivity but not maximal relaxation to ACh in the aorta (Fig. 5A), indicating endothelial dysfunction. Serelaxin had no effect on the sensitivity to ACh, but significantly (*P* = 0.043) increased the maximal relaxation to ACh (Fig. 5A), reversing diabetes-induced endothelial dysfunction in the aorta. To investigate the mechanisms of endothelium-dependent relaxation, ACh response curves were tested in the presence of indomethacin. In the presence of indomethacin, diabetes significantly (*P* = 0.046) reduced the sensitivity but not maximal relaxation to ACh, and serelaxin was able to significantly (*P* = 0.030) restore this effect (Fig. 5B). Furthermore, there was a significant (*P* = 0.0001) reduction in basal NOS levels in the aortae of STZ + Placebo mice, but serelaxin was not able to restore this deficit (Fig. 5C).

### Effects of serelaxin on cardiac remodelling in the LV

**Figure 6:**
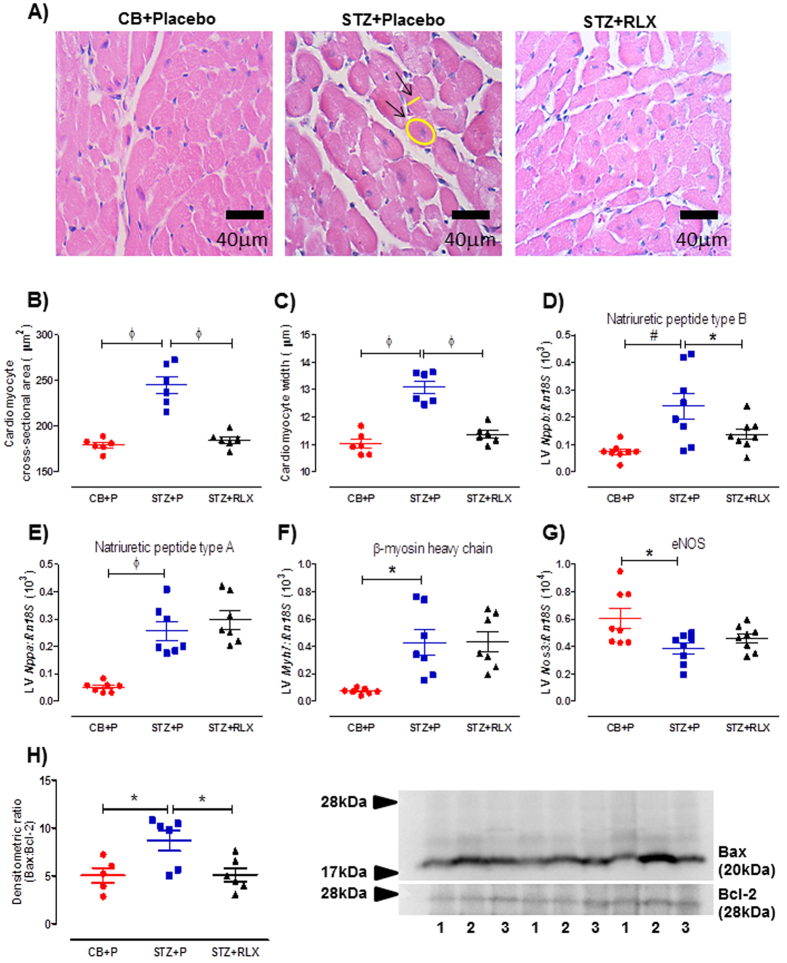
(**A**) Representative and quantification of (**B**) cardiomyocyte cross-sectional area and (**C**) width of H&E-stained cardiomyocytes in the LV of CB+Placebo, STZ+Placebo and STZ+RLX treated mice. Arrows indicate an example of cardiomyocyte cross-sectional area and width measurements. Gene expression of (**D**) natriuretic peptide type B (*Nppb*), (**E**) natriuretic peptide type A (*Nppa*), (**F**) β-myosin heavy chain (*Myh7*) and (**G**) endothelial nitric oxide synthase (*Nos3*) in CB+Placebo, STZ+Placebo and STZ+RLX treated LV. Gene expression is normalised to the reference gene *Rn18S* and presented as mean ± SEM 2^−∆Ct^ values x 10^3^ or 10^4^ (*Nos3*). (**H**) Quantification and representative Western Blot of Bax/Bcl-2 protein expression in the LV of CB+Placebo (denoted by 1), STZ+Placebo (denoted by 2) and STZ+RLX (denoted by 3) treated mice. *n* = 6–8 per group. Significantly (^ϕ^*P* < 0.0001, ^#^*P* = 0.002, *P < 0.05) different to STZ+Placebo.

LV hypertrophy assessed in hematoxylin and eosin (H&E) stained sections revealed a significant (*P* < 0.0001) increase in cardiomyocyte cross-sectional area (Fig. 6A,B) and width (Fig. 6A,C), accompanied by a significant (*P* = 0.002) increase in the anti-hypertrophic natriuretic peptide type B (*Nppb*) mRNA expression (Fig. 6D) in the LV of STZ + Placebo mice compared to CB + Placebo mice. Serelaxin significantly reversed the diabetes-induced increase in cardiomyocyte size (*P* < 0.0001) and *Nppb (P* = 0.05) (Fig. 6A,B,C,D). Additionally, there was a significant increase in natriuretic peptide type A (*Nppa*) (*P* < 0.0001) and β-myosin heavy chain (*Myh7*) (*P* = 0.002), but a significant decrease in endothelial nitric oxide synthase (*Nos3*) (*P* = 0.010) mRNA expression in the LV of STZ + Placebo mice compared to CB + Placebo mice (Fig. 6E,F,G). Serelaxin failed to alter expression of these genes (Fig. 6E,F,G). Apoptosis signalling pathways assessed by Western blot revealed a significant (*P* = 0.023) increase in Bax/Bcl-2 protein expression (Fig. 6H) in the LV of STZ + Placebo mice compared to CB + Placebo mice, and serelaxin significantly (*P* = 0.019) reversed this effect (Fig. 6H).

**Figure 7:**
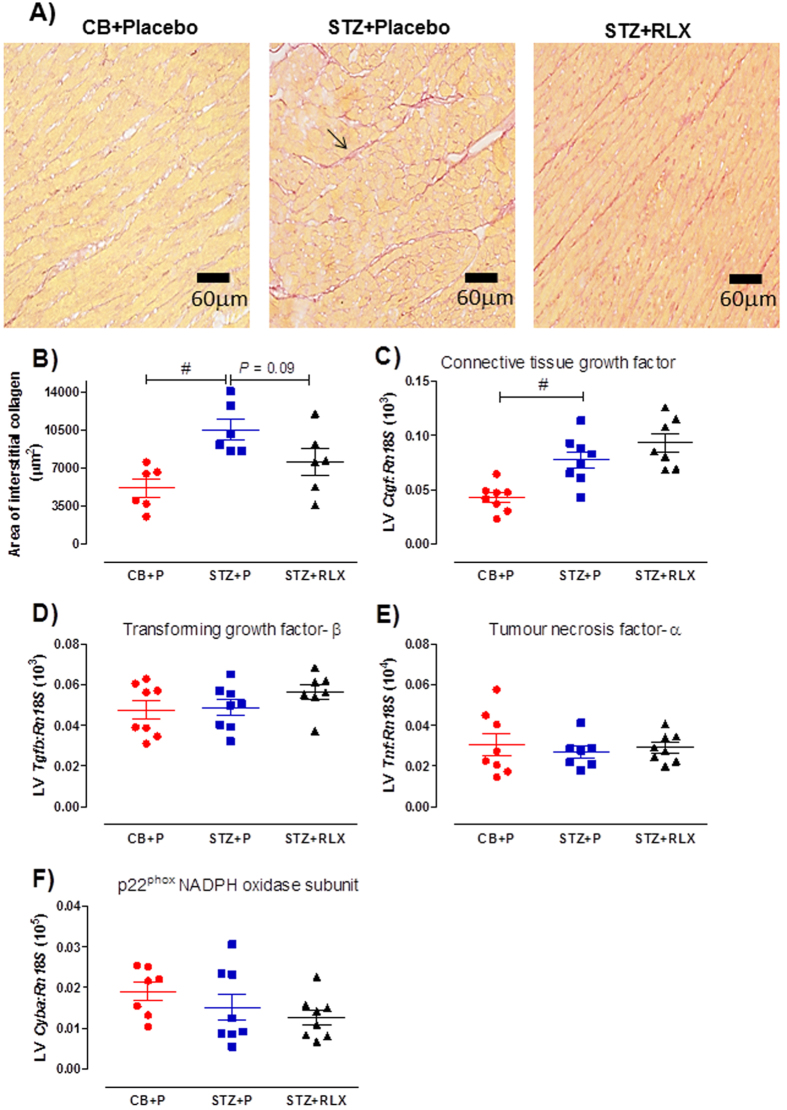
(**A**) Representative and (**B**) quantification of Picrosirius red-stained interstitial collagen in the LV of CB+Placebo, STZ+Placebo and STZ+RLX treated mice. Arrow indicates an example of interstitial collagen. Gene expression of (**C**) connective tissue growth factor (*Ctgf*), (**D**) transforming growth factor-β (*Tgfb*), (**E**) tumour necrosis factor-α (*Tnf*) and (**F**) p22^phox^ NADPH oxidase subunit (*Cyba*) in CB+Placebo, STZ+Placebo and STZ+RLX treated LV. Gene expression is normalised to the reference gene *Rn18S* and presented as mean ± SEM 2^−∆Ct^ values x 10^3^ (*Ctgf, Tgfb*), 10^4^ (*Tnf*) or 10^5^ (*Cyba*). *n* = 6–8 per group. Significantly (^#^*P* < 0.01) different to STZ+Placebo.

Fibrosis assessed in Picrosirius red-stained sections revealed a significant (*P* = 0.006) increase in the area of interstitial collagen (Fig. 7A,B), accompanied by a significant (*P* < 0.001) increase in connective tissue growth factor (*Ctgf*) mRNA expression in the LV of STZ + Placebo mice compared to CB + Placebo mice (Fig. 7C). There was a reduction in the area of interstitial collagen in the LV of STZ + RLX mice compared to STZ + Placebo mice, however this failed to reach statistical significance (*P* = 0.09) (Fig. 7A,B). The inability of serelaxin to significantly reduce interstitial collagen content in the LV of diabetic mice was associated with unaltered *Ctgf* mRNA expression (Fig. 7C). The diabetes-induced increase in interstitial collagen content in the LV was not mediated by changes in transforming growth factor-β (*Tgfb*), tumour necrosis factor-α (*Tnf*) or p22^phox^ NADPH oxidase subunit (*Cyba*) mRNA expression (Fig. 7D,E,F).

### Expression of *Rxfp1* in the mouse LV

Quantitative assessment of relaxin/insulin-like family peptide receptor 1 (*Rxfp1*) mRNA expression in the LV indicated no significant difference between the three treatment groups (Supplementary Fig. 2 A), with an average C_T_ value of 30.

## Discussion

This study demonstrated that serelaxin treatment for two weeks reversed diabetes-induced impairment in endothelial vasodilator function in the mesenteric artery and aorta of STZ-induced diabetic mice. Specifically, serelaxin normalised the contribution of vasoconstrictor prostanoids to endothelial dysfunction and suppressed diabetes-induced hyper-responsiveness to Ang II in the mesenteric artery. It also increased NO-mediated relaxation in both the mesenteric artery and aorta of STZ mice, but had no significant effect on EDH. Favourable effects of serelaxin were not just restricted to the vasculature, but also occurred in the heart because there was marked reduction of diabetes-induced LV cardiomyocyte hypertrophy and apoptosis in serelaxin-treated mice.

In the current study, mice administered with STZ had increased blood glucose levels, HbA_1c_ and plasma osmolality levels after 12 weeks of treatment. Serelaxin treatment for the final two weeks of diabetes did not impact these metabolic parameters. Our findings are similar to a previous study which reported serelaxin had no effects on blood glucose levels in Type 1 diabetic mice^31^. However, in a mouse model of Type 2 diabetes, serelaxin reduced blood glucose levels^30^,^32^. We anticipated a decrease in plasma osmolality in the serelaxin-treated mice because previous studies have shown that shorter durations of serelaxin treatment in rats decreased plasma osmolality^33^. Serelaxin infusion (0.5 μg/d) in late pregnant relaxin-deficient (*Rln*^*−/−*^) mice, which yielded plasma serelaxin levels of approximately 10 ng/mL, also reduced plasma osmolality. Since the osmoregulatory effects of serelaxin are biphasic^33^, it is possible that the longer infusion with a higher concentration of serelaxin in the current study (yielding serelaxin levels of 93 ± 16 ng/mL) might be the reason why there was no effect on plasma osmolality.

Chronic hyperglycaemia selectively impairs endothelial but not vascular smooth muscle function. In mesenteric arteries of diabetic mice, an increased contribution of vasoconstrictor prostanoids to endothelial dysfunction was evident, shown previously to be associated with enhanced oxidative stress^34^. Serelaxin reversed this diabetes-induced endothelial dysfunction by normalising both NO-mediated relaxation to ACh and the contribution of vasoconstrictor prostanoids. The vasoprotective action of serelaxin to enhance NO-mediated relaxation was not limited to the mesenteric bed, but also occurred in the aorta. These data are entirely consistent with the well-established mechanisms of serelaxin’s vascular actions in a variety of blood vessels in healthy animals^19^,^35^. For example, short-term serelaxin administration enhanced BK-evoked NO-mediated relaxation in healthy rat mesenteric arteries via upregulation of basal NOS activity and eNOS protein expression^22^. *Ex vivo* serelaxin treatment also prevented TNF-α induced endothelial dysfunction in the rat aorta by causing phosphoinositide 3-kinase (PI3K)-dependent eNOS dephosphorylation at Thr^495^ and eNOS phosphorylation at Ser^1177^ and Ser633 35. This resulted in increased eNOS activity. Interestingly, although we demonstrated that serelaxin increased ACh-evoked NO-mediated relaxation, there was no significant change in basal NOS activity. Due to limited vascular tissue, we were unable to directly measure eNOS or iNOS activity to confirm this but it is important to note that co-incubation of aortic rings *ex vivo* with serelaxin for three days was also unable to restore the reduction in basal NOS activity induced by high glucose^23^.

A key finding in this study was the ability of serelaxin to normalise the increased contribution of vasoconstrictor prostanoids to endothelial dysfunction in diabetic mesenteric arteries. The potential vasoconstrictor prostanoids in diabetes include TXA_2_, PGD_2_, PGE_2_ and PGF_2α_. Serelaxin reduced the sensitivity to the TXA_2_ mimetic, U46619, and therefore the contribution of this vasoconstrictor prostanoid in diabetic arteries. However, serelaxin increased production of PGI_2_ and the ratio of IP:TP receptors in high glucose treated arteries *ex vivo*^23^. Due to limited vascular tissue, we were not able to investigate the synthesis of TXA_2_ and PGI_2_, or determine expression of IP and TP receptors. Thus, we propose a combination of three possible mechanisms of serelaxin action to normalise the increased contribution of vasoconstrictor prostanoids in the mesenteric arteries of STZ mice.

An unexpected finding was the inability of serelaxin to restore EDH in the mesenteric arteries of diabetic mice. This is in contrast to previous work showing that serelaxin enhanced BK-evoked EDH-mediated relaxation three hours after a single bolus injection^37^. Furthermore, a 10-day serelaxin infusion also increased myogenic reactivity in pressurized cerebral parenchymal arterioles in response to the IK_Ca_ channel inhibitor, TRAM-34^38^. However, both these studies were in healthy animals, so it is possible that serelaxin has limited effects on EDH in diseased vessels.

We further investigated serelaxin’s ability to suppress vasoconstrictor responses in the mesenteric artery. Ang II, the most important component of the renin-angiotensin system (RAS), potently regulates a range of deleterious actions via the angiotensin receptor type 1 (AT_1_R). Diabetes-induced endothelial dysfunction is associated with over-activation of RAS and hyper-responsiveness to Ang II, subsequently increasing oxidative stress via NADPH oxidase (NOX) and eNOS uncoupling^39^,^40^. In the present study, serelaxin suppressed diabetes-induced increases in Ang II-induced contraction. We speculate that these vascular effects of serelaxin are mediated, in part, through heterodimer formation between RXFP1 and AT_2_R, hence counteracting the effects of AT_1_R in mediating vasoconstriction. Heterodimer formation is an important mechanism of action to explain the anti-fibrotic actions of serelaxin in the kidney^41^. Another explanation might be the ability of serelaxin to reduce oxidative stress, leading to the suppression of Ang II-induced vasoconstriction. For instance, serelaxin decreased NOX activity and oxidative markers in the kidney of Ang II-induced hypertensive rats^42^,^43^. Serelaxin also reduced AT_1_R expression in the aorta of *ApoE*^*−/−*^ mice, which was associated with a reduction in superoxide production^44^. In the present study, serelaxin could suppress diabetes-induced hyper-responsiveness to Ang II in a similar fashion. The interaction between serelaxin and Ang II occurs not only in male mice, but also in female mice. The mesenteric artery of pregnant *Rln*^*−/−*^ mice is more sensitive to Ang II during late pregnancy compared to wild-type counterparts^45^.

Prior to our efforts here, the cardioprotective effects of serelaxin in the diabetic heart were largely unknown. Serelaxin treatment for two weeks significantly reversed cardiac stiffness and improved diastolic function in STZ-induced transgenic mRen-2 diabetic rats^46^. In this context, the beneficial effects of serelaxin were associated with its ability to increase matrix metalloproteinase (MMP)-13 and reduce tissue inhibitors of metalloproteinases (TIMP)-1 expression^46^. The anti-fibrotic actions of serelaxin are also attributed to the reduced proliferation of cardiac fibroblasts and type 1 and 3 collagen under high glucose condition^47^, as well as the inhibition of protein kinase C β2 pathway^48^. Consistent with our previous findings^15^, diabetic mice in this study had enlarged cardiomyocyte size, upregulation of hypertrophic genes such as *Nppb, Nppa* and *Myh7* and cardiomyocyte apoptosis^16^. Serelaxin significantly attenuated diabetes-induced hypertrophy, evident across cardiomyocyte size and *Nppb* expression, and prevented further loss of cardiomyocytes via apoptosis. This is in agreement with previous studies that demonstrated serelaxin treatment for two weeks ameliorated cardiac hypertrophy and improved cardiac function in SHRs^25^,^26^. Despite the anti-apoptotic effects of serelaxin in the LV of STZ mice, heart weight did not increase. Diabetes increased size of individual cardiomyocytes which on its own could be anticipated to increase heart weight. At the same time, diabetes decreased the total number of cardiomyocytes in each heart through increased cardiomyocyte loss via apoptosis, which on its own could be anticipated to decrease heart weight. In contrast, administration of serelaxin to diabetic mice prevented both the diabetes-induced increase in cardiomyocyte hypertrophy, and the diabetes-induced increase in cardiomyocyte loss. Although cardiac fibrosis also contributes to heart weight at endpoint, there was no significant impact of serelaxin on most markers of cardiac fibrosis, likely due to the short timeframe of treatment. Overall, there was hence no nett impact of serelaxin on heart weight at study endpoint in diabetic mice. These observations of favourable effects of serelaxin on the phenotype of the diabetic heart, without gross changes in heart weight index, are consistent with other cardioprotective interventions in the diabetic heart, including nitroxyl donors^17^, and both the endogenous antioxidant coenzyme Q10 and the “gold standard” therapy for the cardiovascular complications of diabetes, an angiotensin-converting-enzyme (ACE) inhibitor^16^.

Diabetic mice in the current study presented with increased cardiac collagen deposition and the early pro-fibrotic marker, *Ctgf* expression, but no change in *Tgfb* in the LV indicating the minimal contribution of TGF-β in the initiation of fibrosis. The anti-fibrotic effects of serelaxin have been well documented in a wide variety of experimental models of heart failure^49^, especially through the TGF-β/Smad2 pathway. In our study, we did not observe a marked improvement in LV collagen deposition in serelaxin-treated mice, so it was not surprising that *Ctgf* expression did not change. Although serelaxin marginally (*P* = 0.09) reduced collagen content in diabetic LV, this may be due to enhanced MMP-mediated collagen degradation, as reported in both SHR *in vivo*^25^ and in isolated cardiac fibroblasts *in vitro*^50^. The relative short time frame of serelaxin administration (two weeks) may however have been insufficient for significant blunting of diabetes-induced cardiac fibrosis.

In conclusion, this study demonstrated that *in vivo* serelaxin treatment attenuated diabetes-induced endothelial dysfunction in the mesenteric artery and aorta. This was attributed in part to a serelaxin-mediated reduction in the contribution of vasoconstrictor prostanoids and Ang II to vascular function in the mesenteric artery, and an upregulation of NO-mediated relaxation in both mesenteric artery and aorta. Serelaxin also ameliorated diabetes-induced LV hypertrophy and apoptosis in this mouse model of Type 1 diabetes. Taken together, this study indicates the potential role of serelaxin as an adjunctive agent for the treatment of diabetes-related cardiovascular complications.

## Materials and Methods

### Animal model

This study used male C57BL/6 mice aged 19 weeks old (*n* = 41). All mice were bred and housed under 12 hour day/ night cycle at room temperature of 20 ± 2 °C in the Alfred Medical Research and Education Precinct (AMREP) Animal Centre. The mice were given *ad libitum* access to standard rodent chow (Barastock, VIC, Australia) and water. At six weeks of age, mice were randomly assigned to either control or diabetic groups. Type 1 diabetes was induced in mice by five consecutive daily intraperitoneal injections of STZ (AdipoGen Life Sciences Cat# AG-CN2-0046-G001); 55 mg/kg body weight, in 0.1 M citrate buffer, pH4.5 with overnight fasting^51^. An equivalent volume of citrate buffer was injected into the mice in control group. Eleven weeks after the initial STZ/citrate buffer injections, the mice were further allocated into citrate buffer+placebo (20 mM sodium acetate, pH5.0), STZ+placebo and STZ + serelaxin (Novartis Pharma AG, Basel, Switzerland; 0.5 mg/kg/day) treated groups. Placebo and serelaxin (recombinant human relaxin-2) treatments were administered via sterile subcutaneous osmotic pump (ALZET Model 1002, CA, USA) for two weeks. Blood glucose levels were monitored fortnightly two weeks after the initial STZ/citrate buffer injections using a glucometer (Accu-Chek Performa, Roche, Basel, Switzerland). Mice with blood glucose levels greater than 25 mM were considered diabetic. All experimental procedures were as humane as possible and approved by the AMREP Animal Ethics Committee (E/1535/2015/B) and conform to the Australian Code of Practice & National Health and Medical Research Council guidelines for the care and use of animals for scientific purposes.

### Tissue collection

After two weeks of treatment with either placebo or serelaxin, mice were anaesthetised by a cocktail of ketamine (85 mg/kg) and xylazine (8.5 mg/kg) via intraperitoneal injections followed by cardiac puncture for blood collection. Two μL of whole blood was used to measure HbA_1c_ using a cobas b 101 POC system (Roche, Basel, Switzerland). Blood plasma was used to determine plasma osmolality using a vapour pressure osmometer (VAPRO Model 5600, Wescor Inc, Logan, Utah) and concentrations of serelaxin using a Human Relaxin-2 Quantikine ELISA kit (Cat# DRL200; R&D systems, Minneapolis, MN, USA) following manufacturer’s protocol with detection sensitivity of 4.57 pg/mL. Mesenteric arcade, whole heart and aorta were isolated and immediately placed in ice cold Krebs bicarbonate solution (120 mM NaCl, 5 mM KCl, 1.2 mM MgSO_4_, 1.2 mM KH_2_PO_4_, 25 mM NaHCO_3_, 11.1 mM D-glucose, 2.5 mM CaCl_2_) and then cleared of fat and connective tissues.

### Wire myography

Vascular function was assessed as previously described^21^,^22^ with the following modifications. Briefly, first order mesenteric arteries and abdominal aortae were cut into two mm long rings and mounted on a multi-wire myograph system 620 M (Danish Myo Technology, Aarhus, Denmark) and allowed to stabilise at zero tension for 15 min followed by normalisation. All experiments were performed at 37 °C and continuously bubbled with carbogen (95% O_2_ and 5% CO_2_). Changes in isotonic tension were recorded using Powerlab/ Lab Chart data acquisition system (AD Instruments, Bella Vista, NSW, Australia). Firstly, mesenteric arteries were maximally contracted with high K^+^ physiological salt solution (KPSS, 100 mM), followed by the determination of endothelium integrity. Cumulative dose-response curves to vasoconstrictors such as Ang II (0.1 nM–0.3 μM), PE (1 nM–30 μM), U46619 (0.1 nM–1 μM) and ET-1 (0.1 nM–0.1 μM) were produced to evaluate vascular smooth muscle reactivity. Similarly, cumulative dose-response curves to endothelium-dependent dilator, ACh (0.1 nM–10 μM) and -independent dilator, SNP (0.1 nM–10 μM) were constructed. In order to determine the mechanisms of endothelium-dependent relaxation in the mesenteric artery, responses to ACh were evaluated in the presence and/ or absence of a COX inhibitor, indomethacin (indo, 1 μM), a NOS inhibitor, L-NAME (200 μM), TRAM-34 (1 μM) and apamin (1 μM). ACh-evoked relaxation was analysed using AUC to determine the relative contributions of NO, EDH and PGI_2_-mediated relaxation in the mesenteric artery. Briefly, the contribution of NO was calculated by subtracting AUC of control curve from AUC in the presence of L-NAME. Similarly, the contribution of PGI_2_ was calculated by subtracting AUC obtained in the presence of L-NAME from AUC in the presence of indomethacin + L-NAME. The remaining relaxation after blockade with indomethacin + L-NAME was attributed to EDH-type relaxation.

Aortic rings were maximally contracted with U46619 (1 μM), followed by cumulative dose-response curves to ACh (0.1 nM–10 μM) in the presence or absence of indomethacin (1 μM). In a separate set of experiments, mesenteric artery and aorta were sub-maximally pre-contracted using titrated concentrations of U46619, followed by the addition of L-NAME (200 μM) to determine basal NOS activity. Dose-response curves were fitted to a sigmoidal curve using non-linear regression (Prism version 6.0, GraphPad Software, San Diego, CA, USA) to calculate the sensitivity (pEC_50_) of each agonist. Maximal relaxation (R_max_) was measured as a percentage of U46619 contraction. Maximal contraction (C_max_) was measured as a percentage of KPSS.

### Western Blot

Snap frozen LV were homogenised in ice cold lysis buffer as previously described^21^. Protein concentration of each sample was quantified using a BCA protein assay kit (ThermoScientific). Ninety μg of protein was subjected to SDS-PAGE and Western blot analysis using primary rabbit antibodies for Bax (Cat# 2772, Cell Signalling, Danvers, MA) and Bcl-2 (Cat# 2876) at a dilution of 1:1000 overnight at 4 °C. Protein expression was detected using enhanced chemiluminescence (Western Lightning Plus-ECL Enhanced Chemiluminescence Substrate, PerkinElmer) after incubation with anti-rabbit horseradish peroxidase-conjugated secondary antibody (Cat# 7074, Cell Signalling) in 5% BSA for an hour at room temperature (1:3000). Protein bands were quantified using densitometry and expressed as a ratio of Bax/Bcl-2.

### Quantitative real-time PCR

Total RNA was extracted from frozen LV using TRI Reagent solution (Ambion, Mulgrave, VIC, Australia) as described previously^21^,^22^, resulting in A260/280 values > 1.90. All RNA samples were DNAase treated using DNA-*free*^TM^ DNA removal kit (Ambion, Mulgrave, VIC, Australia) following manufacturer’s protocol to eliminate genomic DNA. First strand complementary DNA (cDNA) synthesis used the SuperScript III cDNA synthesis kit (Invitrogen, Mount Waverly, VIC, Australia) and one μg RNA in a final reaction volume of 20 μL. All RNA samples were reversed transcribed in a single run using a MyCycler Thermal Cycler (BioRad, Gladesville, NSW, Australia). Expression of *Nppb, Myh7, Nppa, Ctgf, Tgfb, Tnf* and *Cyba* was assessed by quantitative PCR using -2^∆CT^ method with ribosomal 18 S (*Rn18s*) as the endogenous reference gene. Mouse-specific forward/reverse primers (GeneWorks, Thebarton, SA, Australia) were generated from GenBank (Supplementary Table 1). Real-time PCR reaction was determined by SYBR green chemistry using the Applied Biosystems 7500 fast real-time PCR system with triplicate samples of 12.5 μL containing SYBR Green PCR Master Mix (Applied Biosystems, Scoresby, VIC, Australia) and 10 μM (gene of interest) or 1 μM (18 S) of primers. For each sample, the mean *Rn18s* C_T_ triplicate value was subtracted from the mean gene of interest triplicate C_T_ value and to normalise gene of interest expression to the reference gene. These normalised data (∆C_T_) were then analysed using the -2^∆CT^ method and presented as mean ± SEM.

In another set of experiments, expression of *Rxfp1* and *Nos3* in the LV was determined on the Applied Biosystems ViiA7 PCR machine (Life Technologies, Mulgrave, VIC, Australia) using Thermo-Fast^®^ 96-well reaction plates (Life Technologies) with triplicate samples of 10 μL containing 2x SensiMix (Bioline, Alexandria, NSW, Australia) and 10 μM of primers and probe. The mean *Rxfp1* and *Nos3* C_T_ value was normalised to the mean *Rn18s* C_T_ value. Mouse specific forward/reverse primers and 6-carbonyl fluorescein-labelled (FAM) Taqman probe (Biosearch Technologies, Novata, CA, USA) were designed to span introns (Supplementary Table 1). Following the determination of *Rxfp1* gene expression by qPCR, the product was run on a 2.5% agarose gel stained with ethidium bromide.

### Histology

A portion of the LV was embedded in paraffin wax, cut into sections of five μm, and mounted on SuperFrost PLUS slides (Menzel-Gläser, Braunschweig, Germany). After an overnight drying at 37 °C, the sections were stained with H&E (Australian Biostain Pty Ltd, Traralgon East, VIC, Australia) for the determination of cardiomyocyte cross-sectional area and width. Cardiomyocyte cross-sectional area and width were determined from the same cell of 100 individual cardiomyocytes per mouse, calculated from cell outlines using Image J. Interstitial collagen area in the LV was examined on the sections stained with 0.1% Picrosirius red solution (Thermo Fisher Scientific, Scoresby, VIC, Australia) and quantified using Image J.

### Materials: drugs, chemicals and reagents

Serelaxin used in this study was kindly supplied by Novartis Pharma AG, Basel, Switzerland. All drugs were purchased from Sigma-Aldrich (Castle Hill, NSW, Australia), except for U46619 (Cayman Chemical, Ann Arbor, MI, USA) and ET-1 (Abcam, Cambridge, MA, USA). They were dissolved in deionised water with the exception of indomethacin which was dissolved in 0.1 M sodium carbonate, TRAM-34 which was dissolved in 100% DMSO and U46619 which was dissolved in 100% ethanol and further diluted in deionised water.

### Data analysis and statistical procedures

One-way ANOVA with Dunnett’s test (vs STZ + Placebo) assessed statistical differences in pEC_50_, R_max_/C_max_, gene and protein expression, cardiomyocyte cross-sectional area/width and interstitial collagen area between groups. Independent t-test assessed statistical differences in pEC_50_ and R_max_ within treatment group in the presence or absence of inhibitor. A level of *P* < 0.05 was considered statistically significant. All data are presented as mean ± SEM, where *n* represents the number of mice per group.

## Additional Information

**How to cite this article**: Ng, H. H. *et al*. Serelaxin treatment reverses vascular dysfunction and left ventricular hypertrophy in a mouse model of Type 1 diabetes. *Sci. Rep.* **7**, 39604; doi: 10.1038/srep39604 (2017).

**Publisher's note:** Springer Nature remains neutral with regard to jurisdictional claims in published maps and institutional affiliations.

## Supplementary Material

Supplementary Information

## Figures and Tables

**Table 1:** Systemic analysis of CB+Placebo, STZ+Placebo and STZ+RLX treated mice.

	CB+Placebo	STZ+Placebo	STZ+RLX
*n*	10	12	12
HbA_1c_ (%)	4.6 ± 0.1^ψ^	11.7 ± 0.6	12.3 ± 0.6
Plasma osmolality (mmol/kg)	306.4 ± 2.3^ψ^	336.6 ± 3.7	332.3 ± 2.4
Body weight (g)	28.6 ± 0.9^ψ^	23.4 ± 0.2	22.9 ± 0.2
Tibia length (mm)	16.2 ± 0.4	16.0 ± 0.2	15.9 ± 0.2
Heart weight/TL (mg/mm)	8.1 ± 0.4^#^	6.4 ± 0.4	6.1 ± 0.3
Left ventricle/TL (mg/mm)	5.8 ± 0.3^ψ^	4.5 ± 0.2	4.4 ± 0.2
Right ventricle/TL (mg/mm)	1.6 ± 0.1^#^	1.2 ± 0.1	1.1 ± 0.1
Atria/TL (mg/mm)	0.6 ± 0.0	0.5 ± 0.1	0.4 ± 0.0

Values are expressed as mean ± SEM; *n* represents number of mice in the group. CB, citrate buffer; STZ, streptozotocin; RLX, serelaxin; HbA_1c_, glycated haemoglobin; TL, tibia length. ^ψ^*P* < 0.0001, ^#^*P* < 0.001, *P < 0.05 (vs STZ+Placebo).
