# Supplementary material for: Serelaxin treatment reverses vascular dysfunction and left ventricular hypertrophy in a mouse model of Type 1 diabetes
Source: Sci Rep. 2017 Jan 9;7:39604. doi: 10.1038/srep39604 (PMC5220363; doi:10.1038/srep39604)
Supplement: Supplementary Information [file srep39604-s1.pdf]

Serelaxin treatment reverses vascular dysfunction and left ventricular hypertrophy in a mouse model of Type 1 diabetes.

Hooi Hooi Ng[1,2], Chen Huei Leo[1], Darnel Prakoso[1,2], Chengxue Qin[2], Rebecca H Ritchie[2], Laura J Parry[1*]

[1]School of BioSciences, The University of Melbourne, Parkville, Victoria 3010, Australia.

[2]Heart Failure Pharmacology, Baker IDI Heart & Diabetes Institute, Melbourne, Victoria 3004, Australia.

Corresponding author:

Prof Laura J Parry, School of BioSciences, The University of Melbourne, Parkville, VIC, 3010, Australia.

Phone +61 3 8344 4379, Fax +61 3 8344 7909

ljparry@unimelb.edu.au

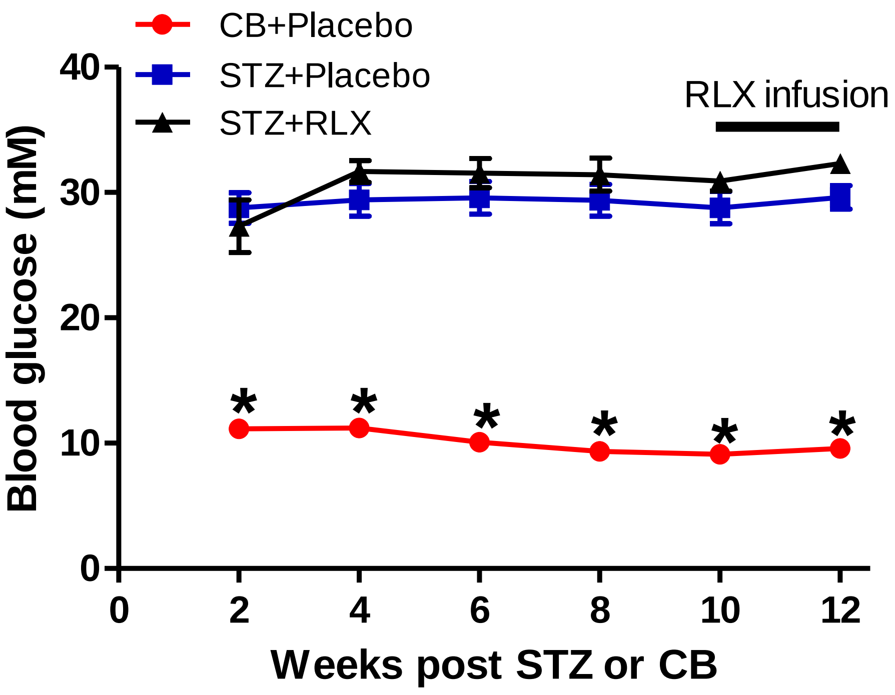

Supplementary Figure 1.

Blood glucose levels of CB+Placebo (●), STZ+Placebo (■) and STZ+RLX (▲) treated mice over the 12-week period after citrate buffer or streptozotocin (STZ) injections. *n* = 10-12 per group. Data are presented as mean ± SEM. *significantly ($P < 0.0001$, 2-way ANOVA, Bonferroni test) different to STZ+Placebo.

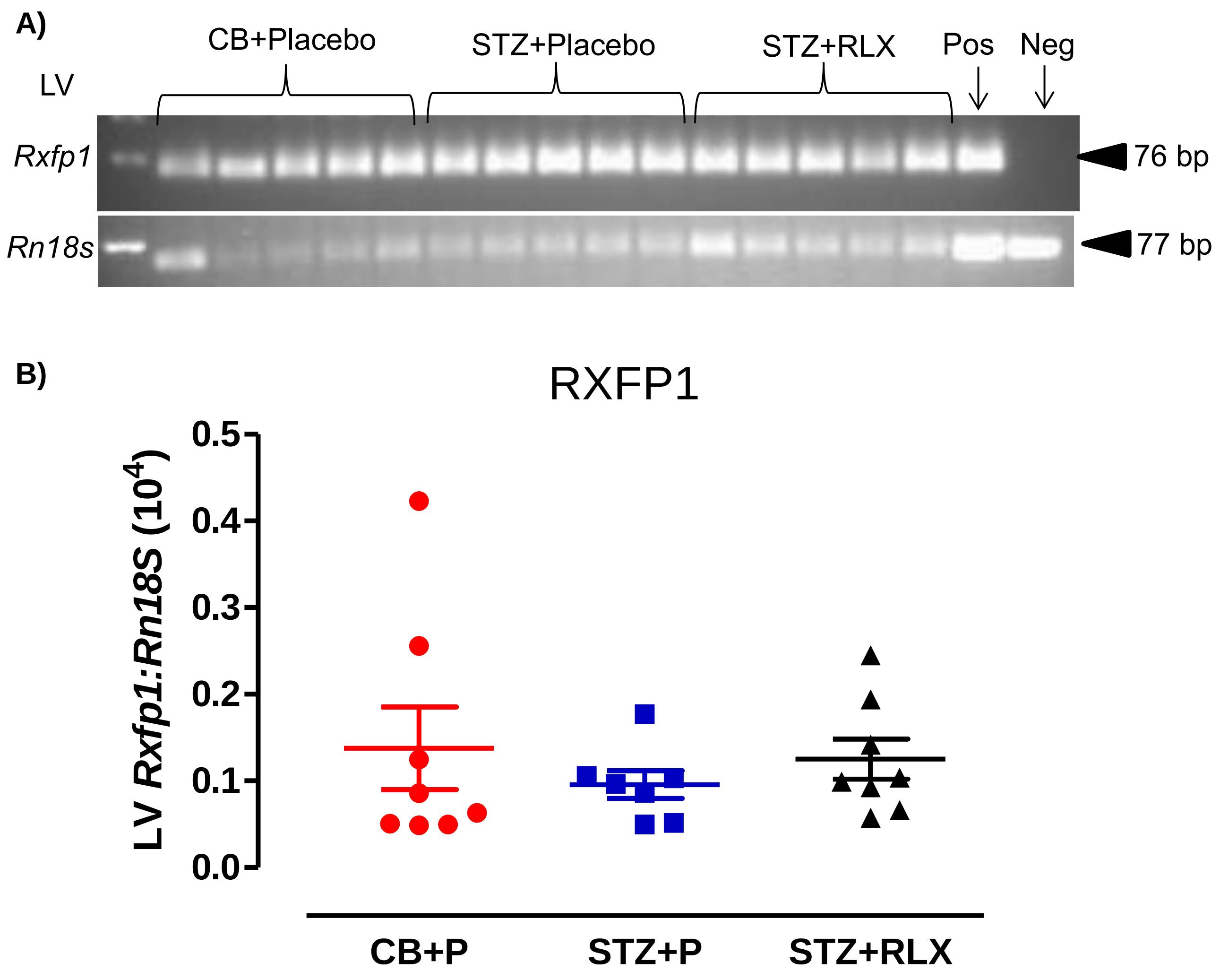

Supplementary Figure 2.

(A) Amplification of relaxin/insulin-like family peptide receptor 1(*Rxfp1*) and *Rn18s* qPCR products on agarose gel electrophoresis and (B) gene expression of *Rxfp1* in CB+Placebo, STZ+Placebo and STZ+RLX treated LV. Positive control for *Rxfp1* was the uterus of mouse on day six of pregnancy. Negative control used the cDNA without SuperScript III in cDNA synthesis (RT negative). Gene expression is normalised to the reference gene *Rn18S* and presented as mean ± SEM $2^{-\Delta Ct}$ values x 10^4. n = 7-8 per group.

Supplementary Table 1. Gene sequences of primers and probes used for quantitative real-time PCR experiments.

Gene		Sequence 5' to 3'
Rn18s	Fwd	TTCGAGGCCCTGTAATTGGA
	Rev	GCAGCAACTTTAATATAGGCTATTGG
Nppb	Fwd	CCTGGCCCATCGCTTCT
	Rev	CATCTGGGACAGCACCTTCA
Myh7	Fwd	TCTCCTGCTGTTTCCTTACTTGCTA
	Rev	GTACTCCTCTGCTGAGGCTTCCT
Nppa	Fwd	TGGGACCCCTCCGATAGATC
	Rev	AGCGAGCAGAGCCCTCAGT
Ctgf	Fwd	TGACCCCTGCGACCCACA
	Rev	TACACCGACCCACCGAAGACACAG
Tgfb	Fwd	TGGAGCAACATGTGGAACTC
	Rev	GTCAGCAGCCGGTTACCA
Tnf	Fwd	CTGTAGCCCACGTCGTAGC
	Rev	TTGAGATCCATGCCGTTG
Cyba	Fwd	AGATCGAGTGGGCCATGTGGGCCAACGAAC
	Rev	CTTGGGTTTAGGCTCAATGGGAGTCCACTG

Gene		Sequence 5' to 3'
Rn18s	Fwd	GCATGGCCGTTCTTAGTTGG
	Rev	TGCCAGAGTCTCGTTCGTTA
	Probe	TGGAGCGATTTGTCTGGTTATTCCGA
Nos3	Fwd	AAGTGGGCAGCATCACCTAC
	Rev	CGGCTCTGTAACTTCCTTGG
	Probe	CCCTGTACCTCAAGACGCTG
Rxfp1	Fwd	GCTTCCACTAACTCCTTTGAGGC
	Rev	CATGCATTGTTTGTGCCGAG
	Probe	AAACTTCCGAATGCGTGGTTGGCTC
